# Determining modulus of elasticity using finite element analysis and non‐destructive testing: Are aquatic animal whiskers stiffer?

**DOI:** 10.1111/joa.14289

**Published:** 2025-06-10

**Authors:** Robyn A. Grant, Charlotte Brassey, Victor G. Goss, Eugene L. Starostin, Tom Allen

**Affiliations:** ^1^ Faculty of Science & Engineering Manchester Metropolitan University Manchester UK; ^2^ School of Engineering London South Bank University London UK

**Keywords:** bend* tests, biomechanics, carnivora, flexural rigidity, pinnipeds, vibrissa*

## Abstract

Approximating the stiffness of biological materials can give important insights into how structures deform and when they may fail. Some samples may be too precious to test to destruction, or too fine to position accurately for conventional material testing, which makes it challenging to obtain approximations of material stiffness. Using two‐dimensional scans, non‐destructive bending tests, and finite element (FE) modeling, we show that we can approximate the modulus of elasticity of samples by fitting FE model data to that of experimental bend tests. We demonstrate our protocol on representative whiskers from three species of Carnivorans, including a terrestrial red fox, semi‐aquatic Eurasian otter, and aquatic phocid grey seal. Grey seal whiskers had the highest approximated modulus of elasticity (0.5–19 GPa), followed by Eurasian otter (0.5–13 GPa) and red fox (0.1–1.5 GPa). We suggest that, as in many other biological structures, adaptations in both the shape and material stiffness of the whisker contribute to how it bends when loaded. Specifically, a larger base radius and higher material stiffness both act to increase whisker flexural rigidity in the aquatic grey seal. This protocol has broad applications in comparative biology and provides a way to determine shape and material stiffness information for various flexible specimen types.

## Graphical abstract

The values of the modulus of elasticity (E) for each species whiskers were obtained when the FE model was aligned to the maximum displacement values of the experiment. Grey seal E values were larger than Eurasian otter, which were larger than red fox. Whisker shape outline examples can be seen for the selected whiskers for grey seals, Eurasian otters, and red fox.

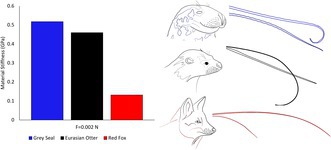

## 1 INTRODUCTION

Understanding and measuring the stiffness of biological materials gives important insights into their evolution and mechanical properties. How stiff a biological structure is, its flexural rigidity (e.g., *EI*), is dependent on two things: (i) the cross‐sectional shape (or second moment of area, *I*); and (ii) the material's resistance to deformation (or modulus of elasticity, *E*). Knowledge of both can help us to better understand how bird bones, feathers, bat wing membranes, and insect cuticles can be light and thin, and yet at the same time be stiff and strong (Bachmann et al., 2012; Khayat et al., 2019; Sullivan et al., 2017; Vincent & Wegst, 2004). The whisker system is another example where flexural rigidity is important and predicted by both whisker shape and material stiffness.

Whisker shape can vary between different whiskers, individuals, and species, including in length, width, curvature, taper, and cross‐sectional shape (Dougill et al., 2023). The resistance of the whisker material to bending will vary with different contributions of composition, including the stiffer cuticle and cortex, and more flexible medulla (Kamat et al., 2024; Quist et al., 2011). The flexural rigidity of each whisker will affect any resulting sensations. Indeed, whiskers transport mechanical signals along the shaft into an innervated follicle, wherein loading information is translated by mechanoreceptors into neural signals and processed in the brain (Campagner et al., 2018; Pammer et al., 2013). Many neural signals in the brain are correlated to the bending moment at the whisker base (Campagner et al., 2018). Therefore, an understanding of how whiskers bend is important to study sensory processing in the mammalian brain (Lucianna et al., 2016).

When estimating flexural rigidity, cross‐sectional shape has been historically easier to measure in whiskers than approximating their modulus of elasticity. Studies have investigated whisker shape, including centerline shape (Starostin et al., 2022), curvature (Luo & Hartmann, 2023; Starostin et al., 2020) and taper (Belli et al., 2017; Dougill et al., 2020; Yan et al., 2013) and all are known to affect bending. However, whisker shape is complex and can vary between individuals and species. For example, Dougill et al. (2020) explored whisker shape in 19 mammalian species and found that terrestrial species had long, thin whiskers, while aquatic pinnipeds had shorter, thicker, and more tapered ones. The same pattern was observed over 24 species of carnivorans, with semi‐aquatic mustelids sitting somewhat intermediate to the aquatic pinnipeds and terrestrial carnivoran species (Dougill et al., 2023). The authors suggested that the larger cross‐sectional area of the whiskers of aquatic pinnipeds caused them to be stiffer. However, material stiffness could also be a contributing factor in stiffening pinniped whiskers. Indeed, the same deflection signal at the whisker base could be achieved via various combinations of material stiffness and cross‐sectional shapes. Teasing out their relative contributions in different species will give us important insights into whisker adaptations and equifinality.

**FIGURE 1:**
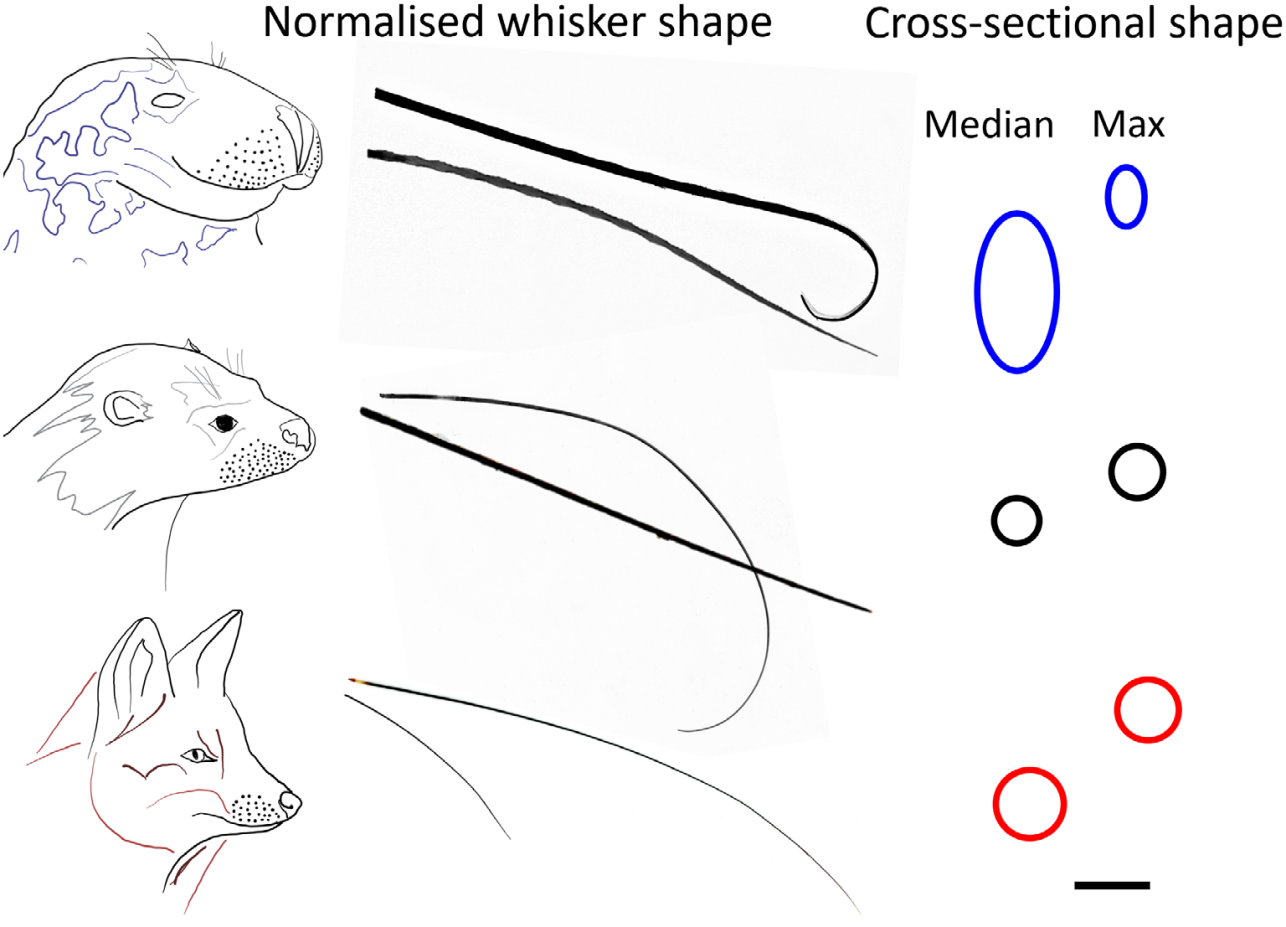
Whisker shapes for grey seals, Eurasian otters and red fox, with the “maximum” whiskers at the top, and the median just below them. Taken from the two‐dimensional photo scans. Maximum whiskers for each species are normalized here to the same length. The whisker base cross‐sectional shape and sizes are also shown (scale bar = 1 mm, for cross‐sectional shapes).

Measuring the modulus of elasticity of a whisker is challenging. Nanoindentation is possible (Herzog et al., 2005; Kamat et al., 2024), but this only measures a discrete point of the whisker. Tensile tests are also possible (Ginter Summarell et al., 2015; Quist et al., 2011), but slender, tapered whiskers are hard to fix, handle, and position (Quist et al., 2011). It can also be challenging to distinguish the effect of material stiffness from that of cross‐sectional shape in a curved, tapered whisker. For whiskers with circular cross sections, this problem can be solved using specialist software that models a whisker under loading (e.g., Elastica 2D, 3D (Huet et al., 2022)). While most mammals have whiskers with circular cross sections, seal whiskers (both phocids and otariids) have non‐circular, oval cross sections (Figure 1). Phocid seal whiskers can also have undulations along their shaft (Figure 1), which influence their flexural rigidity. Ginter Summarell et al. (2015) suggest that such undulations make whiskers more flexible, but we do not know why that might be.

Ginter Summarell et al. (2015) approximated the modulus of elasticity of whiskers from experimental bending tests. For a cantilever experiment where the deflection (*δ*) is measured for different loads at the end (F), they applied simple linear beam theory to give a relationship of the form:

(1) $$F\left(\delta \right)=\left(\frac{3\mathrm{EI}}{L^{3}}\right)\delta ,$$

where *L* is the whisker length, and *I* is the second moment of area. The modulus of elasticity (*E*) can then be determined from the slope (m) of a *F*(*δ*) plot of experimental data, corresponding to Equation (1):

(2) $$E=m\;\frac{L^{3}}{3I}$$

Ginter Summarell et al. (2015) assumed that the whiskers were cylindrical, to approximate I. For the case of a cylinder, with a circular cross‐section, I_C_ can be written as:

(3) $$I_{c}=\frac{\pi }{4}r^{4}.$$

Equation (1) is also derived on the basis of an intrinsically straight, cylindrical whisker, such that both E and I (and their product) are constant. If we assume that E is constant, but I varies from tip to base (due to tapering and undulations), then both Equations (1) and (2) do not hold. This approach does, however, lend support to the idea that if the modulus of elasticity cannot be extracted another way, it can be solved using deflection data and shape information. Indeed, from an experiment, if the whisker's deflection is known (the curvature changes) but the taper and cross‐sectional shape remain constant, by applying Euler‐Bernoulli beam theory, where cross sections along a beam do not change shape during deflection, then it is possible to approximate the modulus of elasticity. One way to do this, especially for complex shapes, is with biomechanical simulations, such as finite element (FE) modelling.

This study will determine whisker modulus of elasticity from an FE model, by fitting the model to experimental bending test data. While FE modeling has been used to approximate modulus of elasticity from modal studies and vibration analyses in human bones (Couteau et al., 1998; Taylor et al., 2002), this is the first to apply such an approach in a comparative morphology context. Establishing and validating such a protocol will enable us to explore the effect of whisker material stiffness (modulus of elasticity) and cross‐sectional shape on bending.

## 2 METHODS

### 2.1 Validation of the approach with an artificial whisker

To validate our method, we used an artificial whisker of a known material that we could approximate. We used a 60 mm long tapered whisker from the robotic Tac Whisker Array 3D printed from nanocure‐RC25, a nanoparticle‐filled material (Lepora et al., 2018; Pearson et al., 2011). We scanned this artificial whisker (Figure S1a) with a photo‐scanner (V600, Epson, Tokyo, Japan). Two‐dimensional (2D) scans are common in whisker shape studies (Belli et al., 2017; Dougill et al., 2020; Starostin et al., 2020) since whiskers tend to lie flat in one plane, and any out‐of‐plane curvature is negligible.

The 2D scan was reconstructed as a three‐dimensional (3D) shape as follows. Shape parameters, including whisker length and major base radius, were available from the scanned image. Scanned jpeg images were imported into computer‐aided design (CAD) software (SolidWorks v. 2022, Massachusetts, USA). The visually approximated centerline of the whisker was manually drawn using the Bezier curve Spline function. A circular cross‐section profile was added normal to the end of this centerline, at the whisker base. Dimensions for the whisker length and base diameter were set based on the 2D scan data. The whisker outline was manually traced in the CAD software using Bezier splines. A Swept Boss/Base was applied to create a 3D shape, with the approximated centerline and outline splines as guidelines and the circular or oval cross‐section profile as the base.

To experimentally measure artificial whisker bending, we clamped its distal (tip‐ward) section to a horizontal surface. Weights were added to the unsupported base section of the artificial whisker, normal to the plane of its intrinsic curvature (See Supplement 2, Figure S2). The length of the clamped distal section remained constant as the applied weights increased. This clamped distal section was 67% of the whisker length (40 mm from the tip). The deflection of the whisker, specifically the displacement of its base relative to its initial position, was measured for two loads. Firstly, with 0.27 g of masking tape (0.002 N) attached to the base section. Secondly, with a 1.27 g mass attached to the masking tape, giving a total of 1.54 g (0.015 N) (Supplement 2, Figure S2). Two loading conditions were chosen to examine whether the material stiffness value was consistent across loads, or whether it was load dependent.

The FE modelling software used was ANSYS Mechanical within ANSYS Workbench (2023, R1, Pennsylvania, US). A non‐linear mechanical quadratic mesh was used. The initial mesh size was set as a tenth of the default mesh. A fixed support was applied to match the experimental testing. A ramped force (0.002 and 0.015 N to match the experiment) was added along an 8 mm section of the whisker base (the width of the attached load). This force bent the whiskers out of their initial osculating plane. The “Large deflections” setting was selected in the software. Step controls were adjusted by time to apply the load slowly and enable convergence of the solution (as recommended in the software manual). An automatic mesh convergence was conducted on the displacement output (<5%).

A material model of isotropic elasticity was created. Varying Poisson's ratio within the material model between 0.3 and 0.4 had negligible influence on displacement. Therefore, Poisson's ratio was set at 0.4. We approximated the whisker material stiffness using only one overall gross measure of the modulus of elasticity. The modulus of elasticity was inputted into the model (as *E*) with a starting value of 3.5 GPa. We then back‐calculated the modulus of elasticity by fitting the maximum displacement output of the FE model to that of the experiment within one decimal place (based on the calliper's resolution).

For the artificial whisker, in both loading conditions (0.002 and 0.015 N), we approximated the modulus of elasticity as 3.6 GPa, which was similar to the mean value of 3.8 GPa (SD = 2.4 GPa) (Supplement 1, Figure S1b) approximated from experimental tests of the same material in the literature (Evans et al., 2013; Rayneau‐Kirkhope et al., 2012) and corresponding datasheet (Technical Data Sheets, Envisiontec, envisiontec.com/materials/, February 2015). The large standard deviation in the experimental value of the modulus of elasticity from the literature is likely due to differences in testing scenarios and specific 3D printing settings between studies. Despite this experimental variation, the fit to the mean value encouraged us to progress to approximating the modulus of elasticity of each animal whisker using the FE model in the same way.

### 2.2 Whisker shape

We chose to model whiskers of a red fox (*Vulpes vulpes*), Eurasian otter (*Lutra lutra*) and grey seal (*Halichoerus grypus*). Five specimens of each species were loaned by National Museums Scotland. These species were chosen to build further on the work of Dougill et al. (2023), who used the same species as terrestrial, semi‐aquatic, and aquatic examples, respectively. The plucking and scanning of the whiskers followed the protocol of Dougill et al. (2023). The work was approved by the Science and Engineering Faculty ethics committee at Manchester Metropolitan University (ID: 364). Scanning to obtain whisker shape was done with the same photo‐scanner as the artificial whisker, with image resolutions of 2 to 8 microns, depending on the overall whisker size. Two measures of whisker curvature, absolute whisker length (mm), width (base radius) and taper gradient (coefficient ω1) were extracted (See Supplement 4, Table S1 for the data, and Dougill et al. (2020) for details), and a Principal Component (PC) analysis was conducted on the data (see Figure 1; Dougill et al., 2023 for PC plots, especially panel 1b for red fox, 1d for Eurasian otter and 1f for grey seal). This analysis was used to get representative whisker shapes for each species to investigate further.

The median (“representative”) and maximum (“extreme”) value of PC1 (42%) was used to select two whiskers for each species (a total of six: 2 whiskers × 3 species, Figure 1), from two different individuals of each species. PC loadings (loadings ≥0.5) indicated that PC1 was mainly explained by whisker length (0.50) base width (ω0: −0.52) and taper (ω1: 0.58), which are the same metrics that vary between aquatic and terrestrial species (Dougill et al., 2020). Inspection of all the whiskers of all five individuals of each species suggested there was a significant difference between whisker base radius (ANOVA: F(2,468) = 97.874, *p* < 0.001; Paired tests: grey seal > Eurasian otter > red fox) in agreement with (Dougill et al., 2023). However, when looking at just the six chosen (median and maximum) whiskers (Figure 1), the grey seal whiskers were thickest at the base, especially along the major (larger) radius, and the Eurasian otter whiskers were thinner at the base compared to the red fox (Figure 1; Supplement 4, Table S1).

As was done with the artificial whisker, each animal whisker was reconstructed as a three‐dimensional (3D) shape from two‐dimensional (2D) scans. Following the centerline approximation, a circular (red fox and Eurasian otter) or elliptical (grey seal) cross‐section profile was added normal to the end of this centerline, at the whisker base. The smallest (minor) base diameter for the grey seal whiskers was measured using calipers. The whisker outline was manually traced in the CAD software using Bezier splines, including for the undulating seal whisker. A Swept Boss/Base was applied to create a 3D shape. Using this technique, the traced cross‐sectional shape was preserved throughout the 3D shape, including normal to the plane of the 2D scan, meaning that taper and undulations occurred across the whole surface of the shape.

### 2.3 Bending tests and simulation

The same experimental bending tests were conducted on the animal whiskers, as well as the artificial whisker. These specific loads (0.002 and 0.015 N) were chosen to ensure the widest possible range of testing loads across the six whiskers. Specifically, the lightest load (0.002 N) caused clear, measurable deformation of the stiffest whisker (2 mm on the grey seal maximum whisker), and the heaviest load (0.015 N) did not deform the most flexible whisker to a near‐vertical position (the red fox maximum whisker). For the Eurasian otter and grey seal whiskers, it was possible to add two more of the 1.27 g masses without them reaching a near‐vertical position. This gave two further loading conditions of 0.027 and 0.039 N, and the data for these can be seen in Supplement 3.

Due to the variation of whisker lengths, the length of the clamped distal section ranged from ~25%–60% of the whisker length (7–31 mm from the tip). It has been suggested that aquatic mammal whiskers may be more flexible once they have been soaked in water (Dougill et al., 2020). Therefore, the test with the lowest mass (0.27 g, 0.002 N) was also repeated after the whiskers had been soaked in water for 30 minutes. There was no significant difference between the wet and mean dry values for whisker bending (Wilcoxon Test: *Z* = –0.105, df = 3, *p* = 0.917), which agrees with Ginter Summarell et al. (2015). Therefore, only the dry conditions were used for comparison with the FE models.

**FIGURE 2:**
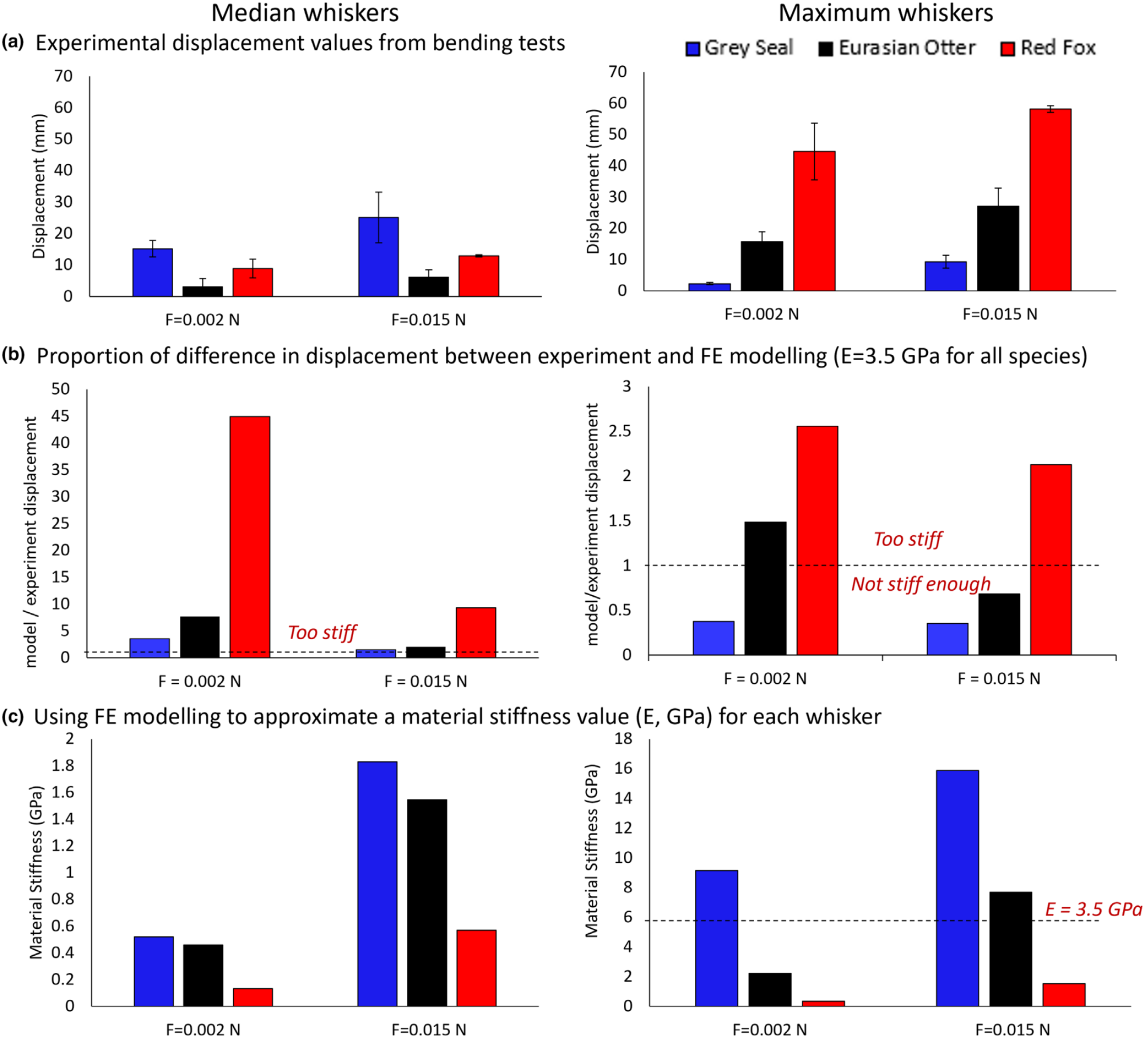
Results summary figure for grey seal (*Halichoerus grypus*), Eurasian otter (*Lutra lutra*) and red fox (*Vulpes vulpes*) whiskers (median on the left and maximum on the right). (a) experimental whisker displacement values from experimental bending tests; (b) proportion of difference between the experimental test and FE model, when the modulus of elasticity (E) in the model was 3.5 GPa for all whiskers. Values above the dashed line (at *y* = 1) indicate that 3.5 GPa is too stiff for these whiskers, whereas values beneath the line indicate that 3.5 GPa is not stiff enough; (c) the values of E for each whisker, when the model was aligned to the maximum displacement values of the experiment. Error bars are standard error.

The FE models were developed in the same way as the artificial whisker version. As with the artificial whisker, the simulated force bent the whiskers out of the plane of curvature (normal to the plane curvature), which was along the smallest (minor) width of the elliptical grey seal whiskers. An automatic mesh convergence was conducted on the displacement output (<5%), and can be seen in Supplement 5, Table S2. Varying Poisson's ratio within the material model between 0.3 and 0.4, the expected range for keratin (Hu et al., 2010), had negligible influence on displacement. Therefore, Poisson's ratio was set at 0.4 again.

For each whisker, the modulus of elasticity was inputted into the model (as *E*) with a starting value of 3.5 GPa, based on approximations for rat whiskers (Hartmann et al., 2003). However, unsurprisingly, the simulated bending displacement values did not match the experimental values (Figure 2). Therefore, we back‐calculated the modulus of elasticity by fitting the maximum displacement output of the FE model to that of the experiment at the two loads (0.002 and 0.015 N) that were given to all species. For the Eurasian otter and grey seal whiskers, the modulus of elasticity was also calculated for the additional loads of 0.027 and 0.039 N, which can be seen in Supplement 3.

### 2.4 Using the model

**FIGURE 3:**
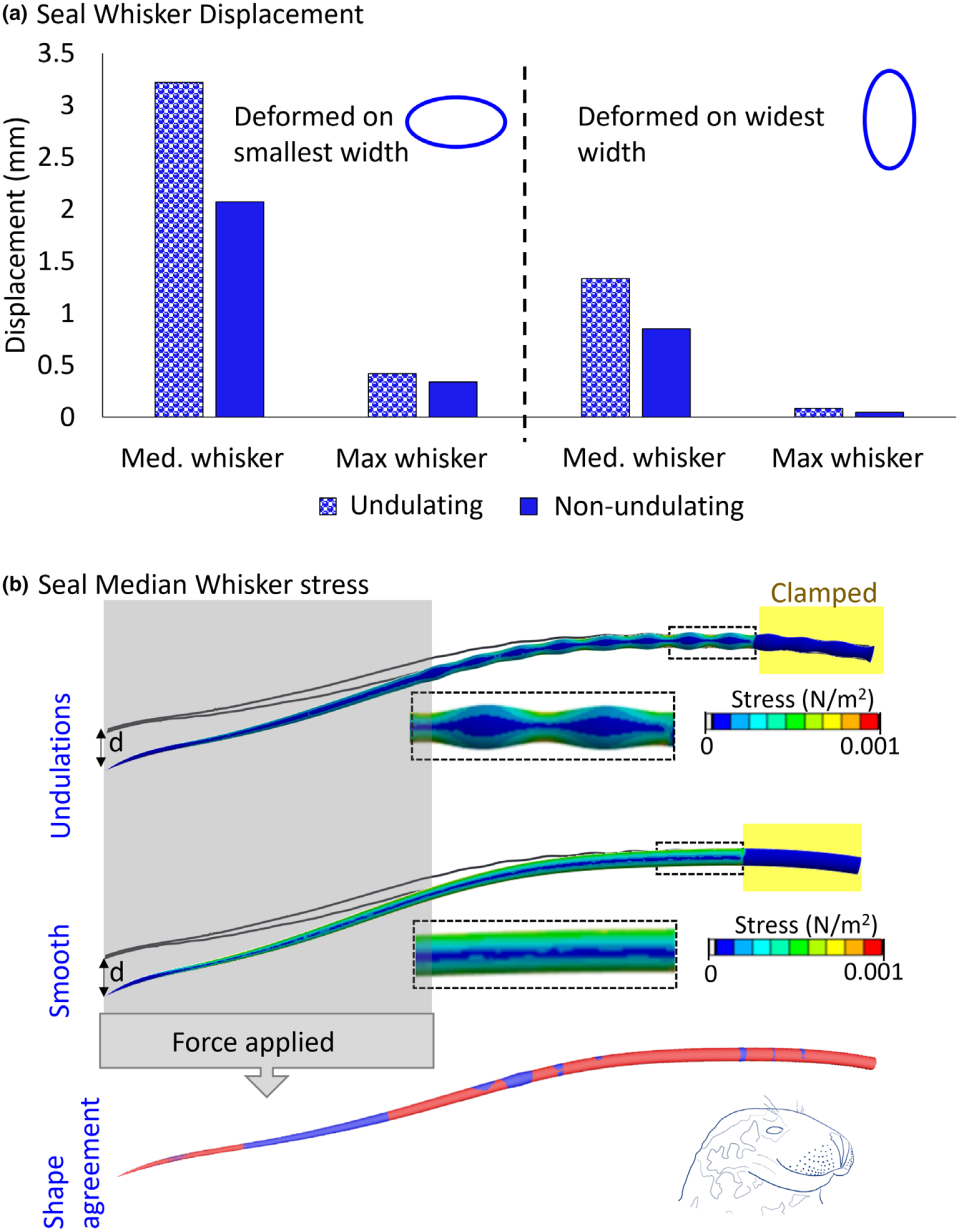
Investigating elliptical cross‐sectional shape and undulations in the grey seal (*Halichoerus grypus*) (a) comparing whisker displacement between an undulating and non‐undulating median and maximum whisker, when a force deformed the whisker on the smallest width and widest width. Maximum displacement was measured at the whisker tip in the direction of the load (i.e., d in panel b, for a load applied on the widest width). (b) An example showing the median grey seal whisker bending along its widest width. The top two plots show the stress distribution from the FEA of the undulating and non‐undulating whiskers. The grey whisker outline shows the undeformed whisker shape from the 2D scan. The dashed lines show the inset area in greater detail. The bottom plot shows the undulating whisker (red) shape aligned to the non‐undulating whisker shape (blue).

To explore the effect of undulations on the pinniped whisker, while controlling for gross shape and total volume, a hypothetical non‐undulating, CAD‐generated whisker was also modeled. This was compared to the original undulating example in simulation using a novel loading condition, with flipped boundary conditions. In this case, a fixed support was added along an 8 mm section at the whisker base and a ramped force of 0.0004 N was added to the distal section (Figure 3b). Force direction was added as above (along the smallest width), as well as normal to this (along the widest width). Maximum deformation and mean strain were examined and compared between both shapes.

## 3 RESULTS

### 3.1 Whisker and species‐specific differences in modulus of elasticity values

When all the whiskers were given the same modulus of elasticity (3.5 GPa), the model did not well‐approximate the bending displacement from the experiment (Figure 2a,b). Looking at Figure 2(b), it is clear that the starting value of 3.5 GPa from the literature was too stiff for all the median whiskers, with the model maximum displacement being much lower than the experimental value, leading to large proportional differences, especially for the red fox (Figure 2b). For the models of the maximum whiskers, the grey seal whisker (at both loads) and the Eurasian otter whisker (at 0.015 N) were not stiff enough, as they displaced more than the experiment. This implies one value cannot adequately capture interspecific and within‐individual variation in whisker modulus of elasticity. Therefore, it was decided to allocate each whisker its own modulus of elasticity value.

Since the modulus of elasticity of the artificial whisker was approximated well using the FE model (Supplement 1, Figure S1), the same approach was used for each individual animal whisker. Different whiskers and species were approximated by different values of modulus of elasticity (Figure 2c). Increasing the force increased the approximated value of modulus of elasticity in all whiskers (Figure 2c). This increase in modulus of elasticity values with increasing forces can also be seen with the two additional loads added to the grey seal and Eurasian otter whiskers in Supplement 3, Figure S3. However, irrespective of whisker (median or max.) or force applied, the ranking of the modulus of elasticity values was the same, with the grey seal having larger modulus of elasticity values than the Eurasian otter, which had larger values than the red fox over these two loads (Figure 2c) (E for grey seal (0.5–16 GPa) > Eurasian otter (0.5–8 GPa) > red fox (0.1–1.5 GPa)).

### 3.2 Cross‐sectional shape and undulations effect bending in grey seal whiskers

Since the grey seal whiskers are both undulating and oval in cross‐section, they were investigated further with our validated model, using the newly established modulus of elasticity values. When a load was added so the grey seal whisker bent on the smallest width, it deformed over twice as much as when it was added on the largest width (Figure 3a). An undulating whisker also deformed more than a non‐undulating whisker, even though both shapes were matched for volume and length (Figure 3b, shape agreement). The stress distribution during deformation shows higher values (i.e., concentrations) at the minima of undulations (Figure 3b). The whisker without undulations had a more even stress distribution along the shaft (Figure 3b).

## 4 DISCUSSION

Using 2D digitization techniques, simple bend tests, and FE modelling, we show that we can approximate the modulus of elasticity of whisker samples. This has many applications, especially if researchers would like to study the mechanical properties of biological specimens in museum collections that are too precious to be tested using destructive material testing or too fine to position accurately for standard material testing. It would be especially useful for highly flexible specimens that can often be more easily measured in bend tests than other material characterization methods, including feathers, spines, hair, and plant material such as stems and branches. Establishing such a protocol has enabled us to explore the effect of the modulus of elasticity and cross‐sectional shape on whisker bending.

We show that using modulus of elasticity values that are specific to each whisker in FE models gives better model approximations of bending displacement than using one value across all whiskers (Figure 2). Our species comparisons show that grey seal whiskers had approximated modulus of elasticity values of 0.5–16 GPa at the two lower forces and up to 19 GPa at the two higher forces. These were larger than those of the Eurasian otter (0.5–8 GPa, and up to 13 GPa at the two higher forces), which were larger than those of the red fox (0.1–1.5 GPa). Theoretically, the modulus of elasticity is independent of loading, assuming linear constitutive relationships. This means we can compare our modulus of elasticity values to those in the literature. For grey seal, these are 3–19 GPa from Ginter Summarell et al. (2015) and 0.5–16 GPa from Kamat et al. (2024), which fall within our range, lending support for our method. However, we do observe that our values of modulus of elasticity are load‐dependent, that the stiffness of the material increases with strain (Figure 2c). Choosing different applied forces may give different values of modulus of elasticity. We selected our forces to represent the widest possible range—from the first detection of a deformation to ensuring that the whiskers did not deform to a near‐vertical position. Therefore, our values of E are likely to be representative for these species.

Despite the modulus of elasticity values varying between loading conditions, we observed the same patterns of modulus of elasticity values between species (grey seal > Eurasian otter > red fox), suggesting that this pattern holds true across different loading scenarios. Non‐linear material models could be developed by experimentally testing whiskers over more loads to generate stress–strain curves. However, we noticed the whiskers were plastic and remained deformed following repeat testing. Indeed, once the elastic limit of a whisker has been passed, the experiments are no longer valid. We applied low forces to prevent permanent deformation of our whiskers, especially the more flexible fox whiskers. This means that our deflection values were relatively small, especially on the stiffer grey seal whiskers, so any errors in measuring whisker dimensions and deflections would have been relatively large. However, we did repeat all experimental testing three times, with all data in good agreement (standard deviation values from the experimental testing can be seen in the Supplementary data file, and standard error values in Figure 2a and Supplement 3, Figure S3a). To improve further upon this, we would suggest that more whiskers, more repeats, and additional loads could be examined, although we recommend using small loads that only cause small whisker deformations, especially if repeat testing is necessary.

The modulus of elasticity values also varied between individual whiskers and are not just simply species‐specific (Figure 2). Shape, including curvature, taper, and undulations, as well as material, including the relative amounts of medulla and cortex of the whisker, will all vary between individual whiskers of the same species (Figure 1a) (Dougill et al., 2020, 2023; Hanke et al., 2010). We propose studying a median whisker as one representative example; this could be a useful approach to studying whisker shape and mechanics. This study is the first where an explicit repeatable protocol has been adopted to select a specific whisker for a more in‐depth study.

Previous studies assumed that the shorter, thicker, and more tapered shape of pinniped whiskers made them stiffer compared to terrestrial species (Dougill et al., 2020, 2023). In this current study, we also noted that the whiskers were shorter and tapered more steeply in the grey seal (Supplement 4, Table S1) than those of the red fox and Eurasian otter (in agreement with Dougill et al., 2020, 2023). In the grey seal, both the elliptical cross section and the undulations affected whisker displacement (Figure 3a). As per Ginter Summarell et al. (2015), we found that undulating whiskers bent more than non‐undulating whiskers. Our hypothetical non‐undulating whiskers were not thicker overall than the undulating whiskers; rather, they had similar mean radii. For example, see Figure 3b, which shows the aligned shapes were visually similar. The larger displacement must, therefore, be due to the lower flexural rigidity (lower *I* with same *E*), specifically at the minima of the undulations. This was supported by the stress distribution map showing concentrations at the minima of each undulation (Figure 3b). Indeed, even small changes in cross‐sectional area will affect the flexural rigidity of the whiskers, since the second moment of area (*I*) has a relationship to the fourth power with the cross‐sectional radius (*r*) (See Equation (3) for an example equation of second moment of area for a cylinder with circular cross‐section, *I*_*C*_). Therefore, it is imperative for the geometry of the shape to be accurate, which is challenging with such slim structures as whiskers. Advancements in scanning technology, such as micro‐computed tomography and synchrotron imaging, will all help with reconstructing accurate 3D shapes in future work.

We agree with previous findings that whisker shape is adapted to the aquatic environment. However, we suggest that, as in many other biological scenarios (Bachmann et al., 2012; Khayat et al., 2019; Sullivan et al., 2017; Vincent & Wegst, 2004), adaptations in both shape and material contribute to whisker stiffness. In grey seals, this has manifested in larger whisker base thickness and modulus of elasticity of the whiskers, making them more resistant to bending in an aquatic environment than Eurasian otter and red fox whiskers. Some of these adaptations in whisker base thickness and material stiffness can also be seen, but to a lesser degree, in Eurasian otter whiskers. The increase in whisker flexural rigidity in aquatic species will likely allow them to position their whiskers precisely during use, such as for hunting, foraging, and navigation in an aquatic environment. This new experimental protocol has given us a useful way to better investigate shape and material stiffness. Applying this approach to more species and examples will give us better insights into the balance that exists between cross‐sectional shape and material properties in predicting flexural rigidity, which has useful applications for studying bending, failure, and strength.

## AUTHOR CONTRIBUTIONS

R.G., C.B., and T.A. contributed to the concept and design of the study. E.S. and R.G. contributed to the acquisition of data and data analysis. R.G. and T.A. interpreted the data. R.G. drafted the manuscript. All authors engaged in the critical revision of the manuscript and approval of the article.

## FUNDING INFORMATION

This work was carried out as part of a Royal Society APEX Grant, APX\R1\211187.

## Supporting information


Data S1.

## Data Availability

The data that supports the findings of this study are available in the supplementary material of this article.
