# Supplementary material for: Determining modulus of elasticity using finite element analysis and non‐destructive testing: Are aquatic animal whiskers stiffer?
Source: J Anat. 2025 Jun 10;247(6):1215–23. doi: 10.1111/joa.14289 (PMC12588165; doi:10.1111/joa.14289)
Supplement: Supplementary file 1 — Data S1. [file JOA-247-1215-s001.docx]

**Supplementary Material**

**Supplement 1: Using the finite element model to approximate the modulus of elasticity in an artificial whisker.**


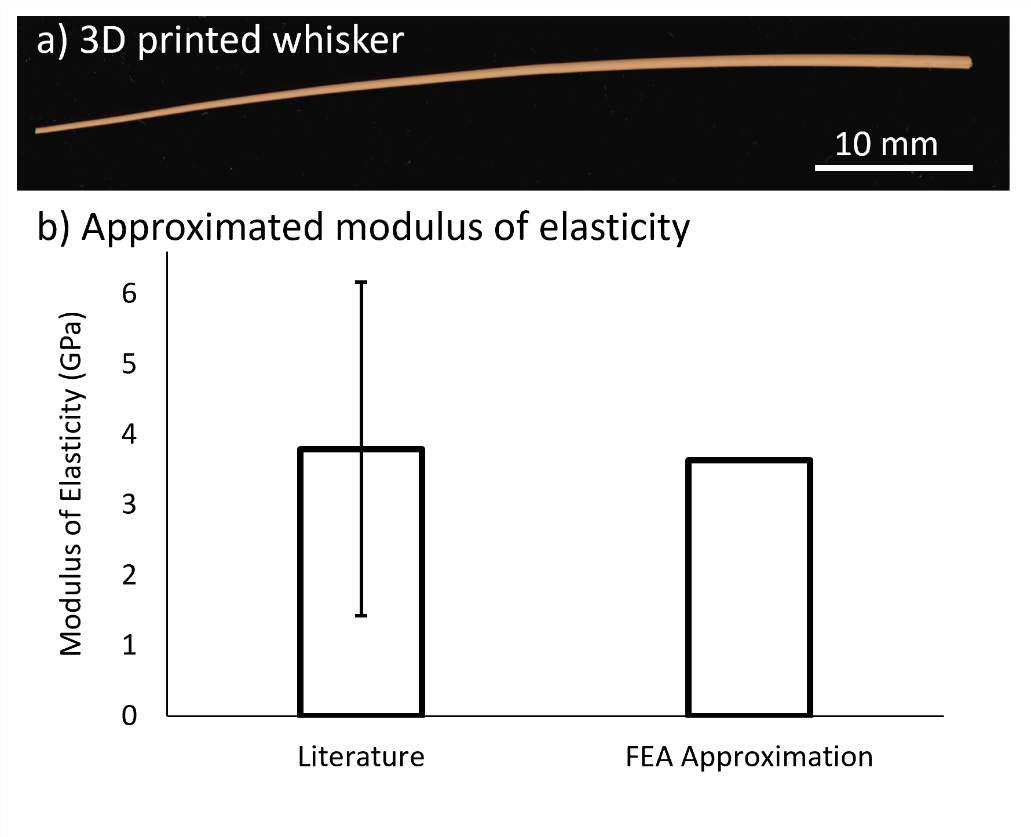


**Figure S1. Approximating the modulus of elasticity in an artificial whisker whisker.** a) Scanner image of the 3D printed artificial whisker, which is curved and tapered; b) the literature data (mean ± standard deviation) compared to an approximation from finite element analysis (FEA). Literature included Evans et al., (2013); Rayneau-Kirkhope et al., (2012) and material datasheets (Technical Data Sheets, Envisiontec, envisiontec.com/materials/, February 2015).

**Supplement 2: Material testing experiments**

**
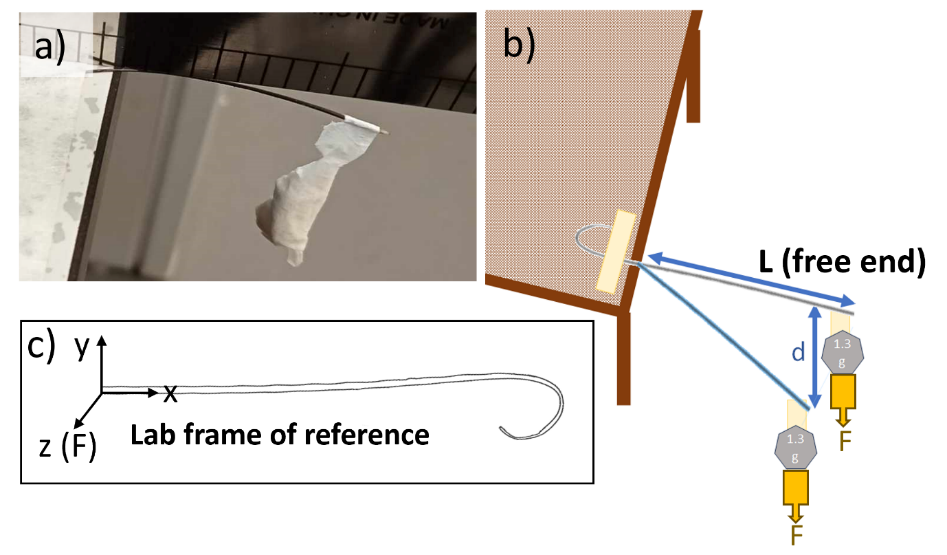
**

**Figure S2. Material testing experiment.** a) photograph of the experiment. The whisker lay flat against a surface, with the thicker base end hanging free, with a load attached with masking tape. b) a diagram of the experiment. The load (force, F) was applied orthogonal to the plane of curvature of the whisker. The free end was hanging with a length (L), and the maximum displacement was measured in the direction of the applied load (F). Panel c shows the laboratory frame of reference, with the load (F) applied in the z direction.

**Supplement 3: Testing additional loads**

**
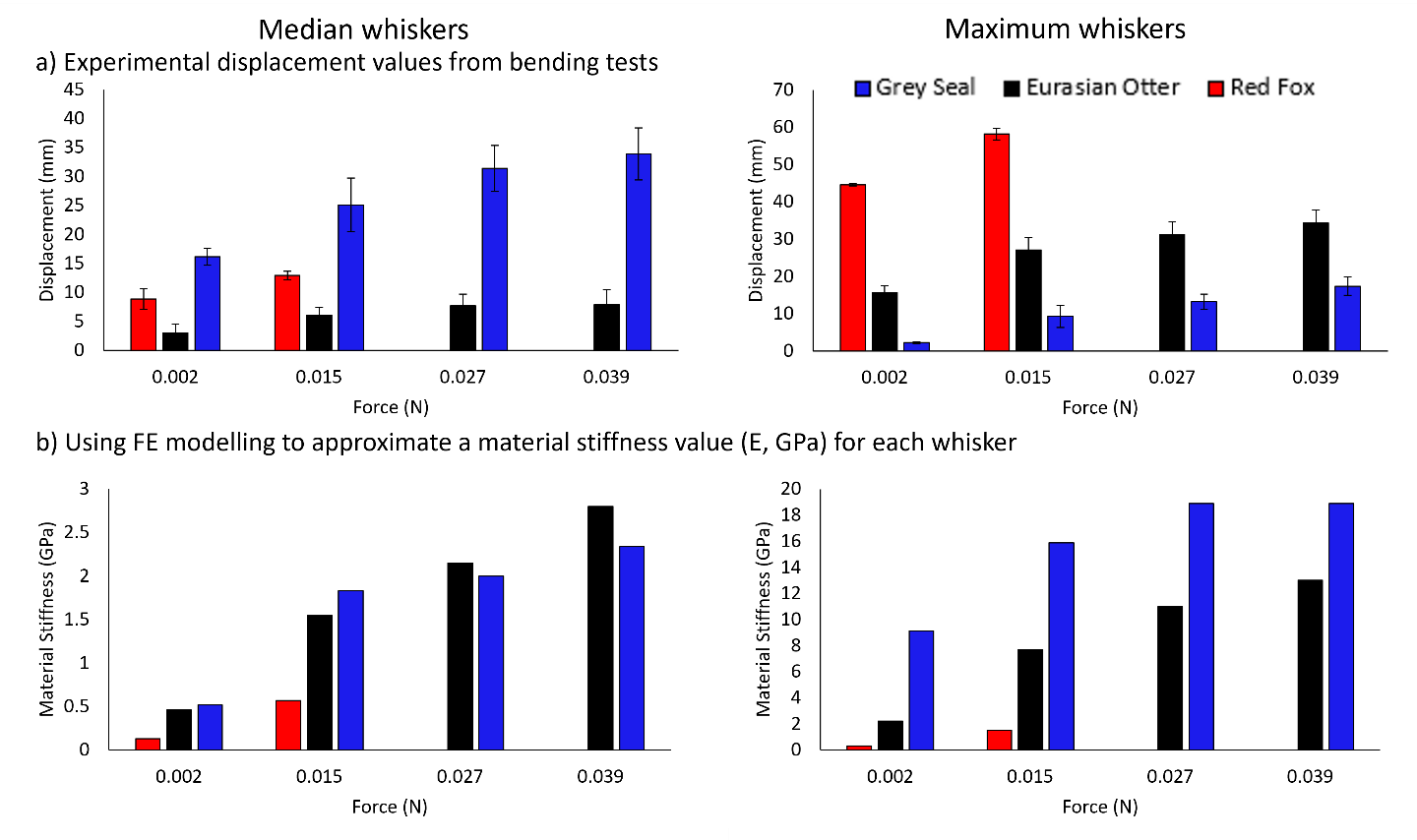
**

**Figure S3 Results summary figure with additional loads of 0.027 and 0.039 N** for grey seal (*Halichoerus grypus*) and Eurasian otter (*Lutra lutra*) whiskers, but not red fox (*Vulpes vulpes*) whiskers as they were too heavy (median on the left and maximum on the right). a) experimental whisker displacement values from experimental bending tests; b) the values of E for each whisker, when the model was aligned to the maximum displacement values of the experiment. I increases with increasing loads, and the fox has the lowest numbers of E, with grey seal whiskers the highest. Error bars are standard error.

**Supplement 4: Whisker shape metrics calculated from scans**

**Table S1. Additional data calculated for these whiskers in Dougill et al. (2023).** Showing two measures of curvature. Refer to Dougill et al. (2023) for how these are calculated.

| **Whisker** | **ID** | **Base radius mm*** | **Length**  **mm*** | **Taper (ω1)** | **Volume**  **mm^3#^** | **Mass**  **mg** | **Curvature (A)** | **Curvature (B)** |
| --- | --- | --- | --- | --- | --- | --- | --- | --- |
| **Red fox Max** | F2 | 0.27 | 83.04 | -0.003 | 17.83 | 7.6 | -1.64 | 0.01 |
| **Red fox Median** | E2 | 0.31 | 54.26 | -0.004 | 12.09 | 1.4 | -.099 | 1.29 |
| **Eurasian otter Max** | D4 | 0.23 | 72.01 | -0.001 | 9.99 | 6.8 | 6.04 | -0.20 |
| **Eurasian otter Median** | E3 | 0.20 | 22.15 | -0.006 | 1.69 | 1.6 | -0.31 | 0.10 |
| **Grey seal Max** | E2 | 0.26 × 0.16 | 51.94 | 0.015 | 13.43 | 20.6 | 7.55 | -2.96 |
| **Grey seal Median** | F3 | 0.52 × 0.35 | 52.05 | -0.007 | 25.37 | 9.3 | -1.34 | 1.00 |

**Supplement 5: Number of mesh elements for each whisker shape**

**Table S2: Number of mesh elements for geometry and after convergence for the 0.002 N force**. NLMA refers to when non-linear mesh adaptation was adopted, so there was no convergence value.

| **Whisker** | **Pre and post mesh convergence** | **No. Elements** |
| --- | --- | --- |
| **Med Fox** | **Geometry** | 12,873 |
|  | **After mesh Convergence** | 22,297 |
| **Max Fox** | **Geometry** | 7,246 |
|  | **After mesh Convergence** | NLMA |
| **Med Otter** | **Geometry** | 14,618 |
|  | **After mesh Convergence** | 21,201 |
| **Max Otter** | **Geometry** | 12,387 |
|  | **After mesh Convergence** | 18,178 |
| **Med Seal** | **Geometry** | 16,925 |
|  | **After mesh Convergence** | 33,709 |
| **Max Seal** | **Geometry** | 76,275 |
|  | **After mesh Convergence** | 81,370 |
